# LAMP-LFD Based on Isothermal Amplification of Multicopy Gene *ORF160b*: Applicability for Highly Sensitive Low-Tech Screening of Allergenic Soybean (*Glycine max*) in Food

**DOI:** 10.3390/foods9121741

**Published:** 2020-11-26

**Authors:** Stefanie M. Allgöwer, Chris A. Hartmann, Clarissa Lipinski, Vera Mahler, Stefanie Randow, Elke Völker, Thomas Holzhauser

**Affiliations:** Division of Allergology, Paul-Ehrlich-Institut (PEI), D-63225 Langen, Germany; stefanie.allgoewer@arcor.de (S.M.A.); chris.a.hartmann@gmx.de (C.A.H.); dr.clarissa.lipinski@gmail.com (C.L.); vera.mahler@pei.de (V.M.); stefanie.randow@pei.de (S.R.); elke.voelker@pei.de (E.V.)

**Keywords:** multicopy gene, rapid test, loop-mediated isothermal amplification (LAMP), lateral flow dipstick/device (LFD), qPCR, food allergy, allergen detection, soybean, *Glycine max*

## Abstract

Soybean (*Glycine max*) allergy can be life threatening. A lack of causative immunotherapy of soybean allergy makes soybean avoidance indispensable. Detection methods are essential to verify allergen labeling and unintentional allergen cross contact during food manufacture. Here, we aimed at evaluating our previously described primers for loop-mediated isothermal amplification (LAMP) of multicopy gene *ORF160b*, combined with a lateral flow dipstick (LFD)-like detection, for their performance of soybean detection in complex food matrices. The results were compared with those obtained using quantitative real-time Polymerase Chain Reaction (qPCR) as the current standard of DNA-based allergen detection, and antibody-based commercial lateral flow device (LFD) as the current reference of protein-based rapid allergen detection. LAMP-LFD allowed unequivocal and reproducible detection of 10 mg/kg soybean incurred in three representative matrices (boiled sausage, chocolate, instant tomato soup), while clear visibility of positive test lines of two commercial LFD tests was between 10 and 10^2^ mg/kg and depending on the matrix. Sensitivity of soybean detection in incurred food matrices, commercial retail samples, as well as various processed soybean products was comparable between LAMP-LFD and qPCR. The DNA-based LAMP-LFD proved to be a simple and low-technology soybean detection tool, showing sensitivity and specificity that is comparable or superior to the investigated commercial protein-based LFD.

## 1. Introduction

Soybean, *Glycine max* (*Fabaceae*), is a commonly consumed legume, used as food ingredient in many cuisines around the world [1]. Regardless of its dietary value, soybean has an allergenic potential. Allergy to soybean is an immunological disorder that is considered a substantial health concern. The prevalence of type-I IgE-related sensitization to soy in a population-based study of adults (aged 20–44 years) in the 13 countries ranged between 0.0% in Iceland and 4.7% in the US, with an overall average of 2.1% for all countries [2]. The consumption of soybean can elicit mild to severe systemic reactions, including anaphylaxis [3,4]. As the second most frequent elicitor of anaphylactic reactions in adults in the German-speaking countries, soybean allergy can potentially be life threatening [3]. Causative therapeutic treatment to cure soybean allergy is not available in clinical routine, so the strict avoidance of allergenic soybean is essential to prevent allergic reactions. To safeguard allergic consumers, many countries require mandatory labeling of soybean when used as an ingredient in prepackaged foods [5]. Nevertheless, hidden allergens, which can be present due to mislabeling or the unintentional cross contact of food products by the use of shared production equipment, may lead to the accidental intake of the offending food [6]. For the protection of allergic consumers, suitable analytical methods are indispensable to allow for the specific and sensitive detection of soybean, with special regard to the verification of labeling requirements and the avoidance of hidden allergens during the food production process. To increase food safety for the great majority of food allergic individuals, detection of allergenic foods should take place at a level where no or, at most, mild allergic reactions are to be expected. The VITAL (Voluntary Incidental Trace Allergen Labeling) expert panel suggested that a dose of 0.5 mg soybean protein, corresponding to 1.25 mg soybean, can trigger allergic reactions in 1% of the soybean allergic population (ED01) [7]. Vice versa, at a level of 1.25 mg soybean, 99% of soybean allergic subjects are considered safe. Thus, to increase safety for the food allergic majority, a method sensitivity of less than 12.5 mg/kg soybean in food is required for a mid-size portion of 100 g.

At present, peptide-based mass spectrometry (MS), protein-based enzyme-linked immunosorbent assays (ELISA) and nucleic acid (DNA)-based quantitative real-time polymerase chain reaction (qPCR) are most commonly used for soybean detection in food [8]. However, MS, ELISA and qPCR are laborious procedures, requiring professional expertise and some level of sophisticated instruments, making them unsuitable as simple on-site detection methods alongside the food manufacturing processes.

Here, the qualitative, rapid and simple DNA-based loop-mediated isothermal amplification (LAMP) can constitute an alternative method for DNA-based allergen detection. According to a survey of proficiency testing within six years, DNA-based analysis appears to be more robust for soybean detection than protein-based assays [9]. Thus, LAMP could complement or replace existing commercial protein-based rapid methods for soybean detection. LAMP is based on a set of three primer pairs, targeting eight different fragments of the target sequence and the strand displacement activity of the *Bst* polymerase. This strand displacement activity eliminates the need for a denaturation step and, thus, of a thermal cycler, and the amplification reaction can be conducted under isothermal conditions at 60–65 °C, which makes this method highly cost- and time-efficient, as well as easy-to-perform [10,11]. A simple thermostatically controlled water bath, heat block or oven is the only equipment required [10,11].

The amplification products can be detected via various methods, i.e., agarose gel electrophoresis (AGE) [12,13,14], using intercalating fluorescent nucleic acid dyes [13,14,15], by turbidity formation [16], colorimetric reagents [15] or a lateral flow device (LFD) [17]. The LFD is a fast and simple way to detect successful amplification. Here, labeled primers result in labeled amplification products, which are bound by antibodies on the test stripe, and analysis does not require special instruments [17]. In addition, the use of multicopy genes may increase the detection sensitivity of DNA-based assays [8].

Previously, we described LAMP primers for the specific and sensitive detection of soybean using a multicopy gene, the mitochondrial open reading frame 160b (*ORF160b*) of soybean, as the specific target for DNA amplification in a range of soybean cultivars. Moreover, we showed that an LFD-like detection was superior to AGE, turbidity, or fluorescence detection regarding distinct signal visualization that is independent from laboratory instruments [18].

In the present study, we aimed to validate the previously described *ORF160b* LAMP-LFD regarding its sensitivity for the detection of accurately defined amounts of soybean in three model food matrices, and in various processed soy products. The method applicability was investigated in a range of retail samples, potentially containing soybean according to the ingredient list or due to precautionary allergen labeling (PAL), such as “may contain …”. The performance of the qualitative *ORF160b* LAMP-LFD assay was further compared with that of qPCR, the current standard in DNA-based allergen detection [8], as well as with two commercially available protein-based LFDs, which are examples of state-of-the-art technology for simple and rapid qualitative allergen detection [19].

Based on our thorough validation in various food matrices, we conclude that this LAMP assay, targeting a multicopy mitochondrial gene, allows LFD-like detection of soy that is as reliable as qPCR, and at least as sensitive as selected commercial antibody-based LFD.

## 2. Materials and Methods

### 2.1. Plant Materials and Retail Foods

Single component foods (Table S1) were purchased at local retailers or were donations by German seed breeding companies. Processed soy products, i.e., texturized vegetable protein (TVP), flakes, protein concentrate, defatted flour, semolina and tofu, were kindly provided by Dr. Wolfgang Weber, the Institut für Produktqualität (ifp, Berlin, Germany), or purchased at a local retailer. Ingredients for incurred sausages, commercial chocolate, as well as the commercial instant tomato soup and commercial retail foods were purchased at local supermarkets. The samples were ground using an analytical mill (M20, IKA Labortechnik, Staufen, Germany), a CryoMill (Retsch, Haan, Germany) or a knife mill (Grindomix GM200, Retsch, Haan, Germany) and stored at −20 °C until further use.

### 2.2. Soybean Incurred Food Matrices

For investigation of method sensitivity and potential matrix interference on soybean detectability, self-made boiled sausages, commercial dark chocolate (Alnatura, Darmstadt, Germany) and instant tomato soup (Cenovis, Radolfzell, Germany), incurred with known amounts of soybean (commercial whole yellow soybeans, Schoenenberger^®^ Hensel^®^, Magstadt, Germany), were prepared. Soybeans were separately homogenized using a knife mill (Grindomix GM200, Retsch). Prior to preparation of the incurred matrices, the sausage ingredients, the chocolate, and the instant tomato soup were tested soy negative using the *lectin* qPCR as described below. Matrices were incurred at levels of 10^5^ (10%) mg, 10^4^ (1%) mg, 10^3^ (0.1%) mg, 10^2^ (0.01%) mg, 10^1^ (0.001%) mg and 10^0^ (0.0001%) mg soybean per kg matrix by a repetitive serial dilution of incurred matrix in blank matrix (details as follows).

#### 2.2.1. Sausages

Pork haunch, pork belly with rind and table salt were purchased at local supermarkets. Three kg of pork meat was minced and mixed with 1% table salt for 10 min. Nine proportions of weight of the minced sausage ingredients were mixed with one proportion of weight from ground soybeans, and the mixture was then homogenized under liquid nitrogen to form a fine powder (IKA M20, IKA Labortechnik, Staufen, Germany). Lower levels of incurred soybean were obtained out of previously incurred matrix by serial repetition of the protocol with the blank matrix. The incurred minced sausage mixture was filled in natural pork gut and boiled in a water bath at 75 °C for 35 min. Sausages with various levels of incurred soybean were stored at −20 °C until use.

#### 2.2.2. Chocolate

Chocolate spiking was previously published [19]. Briefly, ground soybeans were mixed 1:10 (*w*/*w*) with chopped chocolate, melted (42 °C, 10 min), and stirred. The hardened sample (−20 °C, 20 min) was ground under liquid nitrogen, as described above. Melting, grinding and hardening was repeated once. Frozen chocolate with incurred soybean was chopped in a knife mill (Grindomix GM200, Retsch, Haan, Germany). Lower levels of incurred soybean were obtained by serial repetition, as described above.

#### 2.2.3. Instant Tomato Soup

Nine proportions of weight of instant tomato soup were mixed with one proportion of weight from ground soybeans, and the mixture was then homogenized under liquid nitrogen to form a fine powder (IKA M20, IKA Labortechnik, Staufen, Germany). Lower levels of incurred soybean were obtained from a previously incurred matrix by serial repetition of the protocol with the blank matrix.

### 2.3. Commercial Protein-Based LFD

Two commercial antibody-based LFD tests, termed LFD1 and LFD2, were applied, according to the manufacturers’ instructions (RIDA^®^Quick Soya R7103, R-Biopharm, Darmstadt, Germany; AgraStrip^®^ Soja, Romer Labs, Butzbach, Germany). In principle, one sample replicate was taken, extracted and measured as a single replicate. Each LFD test was visually inspected and assessed with a positive outcome if both the test line and control line were color-stained uninterruptedly. For analysis using LFD1, 1 g of ground sample and 7.5 mL of extraction buffer, pre-heated to 60 °C, were shortly mixed and incubated for 10 min at 100 °C in a water bath. After chilling on ice, the mixture was filtered through 5 µm and 1.2 µm syringe filters (Sartorius AG, Göttingen, Germany). Each five drops of Conjugate 1 and Conjugate 2, as well as 150 µL sample extract, were mixed in a 5 mL reaction tube and incubated at room temperature. After 5 min incubation, the test strip was placed into the solution for read-out after another 5 min. For analysis using LFD2, 200 mg of ground sample was added into the provided reaction tube and filled up with extraction buffer up to the marking. After mixing the solution for one minute by hand, 12 drops of the mixture were transferred into a provided incubation tube. After 15 s of mixing, the mixture was incubated at room temperature for another 5 min. Subsequently, the test strip was placed into the solution for exactly 5 min. Here, the manufacturer explicitly points out to immediately evaluate the results after 5 min incubation, since longer incubation times could lead to the development of false positive results.

### 2.4. DNA Extraction and Purification

The DNA extraction was performed as previously described [18]. Briefly, 100 mg of ground sample powder was extracted in 1.4 mL cetyltrimethylammonium bromide buffer (55 mM CTAB, 1400 mM NaCl, 20 mM EDTA, 100 mM Tris, pH 8.0) and 20 μL of proteinase K at 65 °C for 60 min. The supernatant was clarified using chloroform. DNA was precipitated using chilled isopropanol in the presence of mussel glycogen, and washed with 70% ethanol. The dried DNA pellet was resolved in 100 μL of TE buffer (10 mM Tris HCl, 1 mM EDTA, pH 8.0) overnight, and subsequently purified and eluted in 50 µL EB buffer using the QIAquick PCR Purification Kit (Qiagen, Hilden, Germany). The DNA concentration and purity were measured by UV absorption at 260 nm and the ratio at 260 nm/280 nm, respectively.

### 2.5. Oligonucleotides

LAMP primers (F3, B3, FIP, BIP, LoopF and LoopB), targeting the *ORF160b* sequence (GenBank acc. No. JX463295.1), were previously developed and published by the authors [18]. For LFD detection, primers FIP and LoopF of both LAMP primer sets were 5′-biotin-labeled and 5′-FITC-labeled, respectively. Primers and probes for soybean detection using *lectin* qPCR were used as described in the official collection of test methods according to German Food and Feed law [20]. Allmann et al. [21] previously published universal eukaryotic PCR primers TR03/TR04. All oligonucleotides were synthesized by biomers.net GmbH (Ulm, Germany). Primer sequences are displayed in Supplementary Materials (Table S2).

### 2.6. Eukaryotic qPCR

To exclude the presence of inhibitors that possibly lead to false negative results, the amplifiability and quality of the extracted DNA was verified with previously published universal eukaryotic PCR primers TR03/TR04 (Table S2) targeting the 18S coding region of the rRNA [21]. The eukaryotic qPCR was performed as previously described [18] using a MX 3005P™ qPCR detection system (Stratagene, San Diego, CA, USA). A total of 5 μL of 1:10 diluted sample DNA or sterile water as a negative control was added to 20 μL of reaction mixture. A reaction was considered positive if fluorescence exceeded the set threshold fluorescence.

### 2.7. Generation of Plasmid Standards for Lectin qPCR

A specific part of the *lectin* gene was amplified by conventional PCR. The 25 µL reaction mixture contained 5 μL of extracted yellow soybean DNA, 1× Taq DNA Polymerase PCR Buffer (Invitrogen), 200 μM each dNTP including dTTP (Carl Roth, Karlsruhe, Germany), 5 mM MgCl_2_ (Fisher Scientific GmbH, Schwerte, Germany), 0.25 mg/mL BSA solution (Sigma Aldrich, St. Louis, MO, USA), each 300 nM forward and reverse *lectin* primer, and 0.025 U/µL Platinum Taq DNA polymerase (Invitrogen). After thermal cycling (initial denaturation at 95 °C for 10 min, followed by 45 cycles at 95 °C for 15 s and 60 °C for 1 min), PCR products were separated in a 3% (*w*/*v*) high resolution agarose gel (Carl Roth, Karlsruhe, Germany) using “Rapid Agarose Gel Electrophoresis System” (RAGE system, Cascade Biologics, Portland, OR, USA), according to the manufacturer’s instructions. The 81 bp PCR products, elucidated with ethidium bromide (0.75 μg/mL) using UV light (312 nm) on transilluminator (Intas Gel Jet Imager, Intas Science Imaging Instruments GmbH, Göttingen, Germany), were excised from the gel and purified by using the Qiaquick Gel Extraction Kit (Qiagen) according to the manual. The purified PCR amplicons were ligated in linearized pJET1.2/blunt vectors according to the manufacturer’s protocol (CloneJET PCR Cloning Kit, Fisher Scientific GmbH, Schwerte, Germany). Plasmids were transformed into chemically competent *E. coli* “One Shot^®^ TOP10” cells (Invitrogen) and positive clones were amplified in PCR according to the manufacturer’s protocol using the REDTaq^®^ ReadyMixTM PCR Reaction Mix (Sigma Aldrich, St. Louis, MO, USA). Positive PCR amplicons were analyzed by RAGE and clones containing plasmids with correct amplicon size were selected for overnight culture. Plasmid DNA was isolated using the QIAprep Spin Miniprep Kit (Qiagen), according to the manufacturer’s protocol. All cloning was verified by sequencing (Eurofins MWG Operon, Martinsried, Germany) and plasmids containing the 81 bp *lectin* amplicons were 10-fold serially diluted from 2 × 10^5^ to 2 × 10^0^ copies per µL, after UV quantification (Implen NP80 NanoPhotometer).

### 2.8. Lectin qPCR

Primers and probes for soybean detection using the *lectin* qPCR according to the German Food and Feed law [20] are shown in Table S2. The probe was labeled with the reporter dye ROX at the 5′ end and the quencher BBQ-650 at the 3′ end. The mastermix of the qPCR method comprized 1× Taq DNA Polymerase PCR Buffer (Invitrogen, Life Technologies, Carlsbad, CA, USA), each 200 μM dATP, dCTP, dGTP and 400 µM dUTP (Carl Roth), 5 mM MgCl_2_ (Fisher Scientific GmbH, Schwerte, Germany), 0.25 mg/mL BSA solution (Sigma Aldrich), each 0.3 µM forward and reverse primer, 0.2 µM probe, 0.01 Units/µL Uracil-N-glycosylase (UNG, Jena Bioscience, Jena, Germany), 0.025 Units/µL Platinum *Taq* DNA polymerase (Invitrogen), 5 μL sample DNA and sterile water that was added to a final volume of 25 μL. The protocol consisted of the following steps: First, one cycle of UNG-mediated cleavage of potential desoxy-uracil-containing amplicons from previous PCR runs at 50 °C for 2 min, followed by one cycle of initial template denaturation and *Taq*-activation at 95 °C for 10 min and followed by 45 cycles of denaturation at 95 °C for 15 s, and subsequent annealing with polymerization at 60 °C for 1 min. qPCR experiments were performed on a MX 3005P™ real-time PCR cycler (Stratagene, via Agilent Technologies Sales & Services GmbH & Co., KG, Waldbronn, Germany). The fluorescence in qPCR experiments was read using the ROX channel, and the threshold cycle CT was calculated collectively for all samples of one run by the software program MxPro (MxPro-Mx3005P v4.10 Build 389, Schema 85, Stratagene) using the manual threshold setting at a suitable fluorescence level. A standard curve was generated by analysis of six 10-fold dilutions of the reference target plasmid (10^6^ to 10^1^ copies per reaction) to serve as a positive control for reproducibility of sensitivity. For NTCs, qPCR mixtures contained 5 μL of sterile water instead of DNA template. A reaction was considered positive if fluorescence exceeded the set threshold fluorescence.

### 2.9. ORF160b LAMP-LFD

Isothermal LAMP reactions (50 min, 62 °C), targeting the *ORF160b* gene, and subsequent LFD-like detection of dual-labeled amplification products, using a commercial LFD dipstick (Milenia HybriDetect, Milenia Biotec GmbH, Gießen, Germany), were exactly carried out as previously described [18], in a total reaction volume of 25 μL containing the reaction mixture and 5 μL template DNA. For no template controls (NTCs), 5 μL of sterile water instead of DNA template were added. A reaction was considered soy positive if both control and test line developed visible bands according to the LFD manufacturer’s instructions. A negative test line, i.e., soy negative result, was valid if the control line visibly developed.

### 2.10. Replicate Analysis of Food Samples

The analysis of specificity of the *ORF160b* LAMP-LFD assay was previously described in detail [18]. The specificity of the *lectin* qPCR was not investigated. Both commercial protein-based LFDs were tested against 18 species (including soybean) commonly used as food ingredients (Table S1). The number of replicates (*n* = 1) was chosen according to the LFD manufacturers’ instructions. Samples were investigated using 1:10 diluted extracts (Table 1). For the analyses of differently processed soybean products, all protein extracts and DNA extracts were analyzed at 1:10^4^ dilution using DNA-based (LAMP, qPCR) and protein-based methods (LFD), respectively. The total number of replicates, based on the number of replicate extracts and reaction replicates thereof, are displayed in Table 1. For the determination of method sensitivity in incurred food matrices, and the investigation of applicability in real-life retail food samples, all protein extracts and DNA extracts were analyzed undiluted using DNA-based (LAMP, qPCR) and protein-based methods (LFD). The total number of replicate analysis, based on the number of replicate extracts and reaction replicates thereof, are displayed in Table 1.

### 2.11. Statistical Analysis

The qualitative results of the analysis of soy incurred food matrices and selected retail foods, as obtained with the different soybean detection methods, were statistically analyzed using MS Excel 2016 and SigmaPlot 14.0 (Systat Software GmbH, Erkrath, Germany). We performed McNemar’s Test, similarly as previously described [22], to analyze if observed differences of results of investigated samples were statistically significant between methods. The results of two compared methods were arranged binary (positive: 1; negative or doubtful: (0) and counted. Differences were considered as statistically significant with χ^2^ (Chi-square) larger than the critical value (3.841459) and *p* < 0.05 for 95% probability and one degree of freedom.

## 3. Results

### 3.1. Specificity of the Methods

Proteins from 18 different single component foods (Table S1) were extracted and analyzed at 1:10 dilution (equivalent to 10% of a food), using two commercial LFD tests. A positive result was obtained with soybean, but none of the other foods tested. The *ORF160b* LAMP-LFD assay, as previously published [18], showed high specificity for soybean with no observed cross-reactions to other commonly used food ingredients. The *lectin* qPCR was not investigated for specificity, because of its proven long-term use as a detection method for soybean as an allergen and as a reference gene for genetically modified soybean alike [20].

### 3.2. Detectability of Soy Products

Traditional soy foods made from soybean, such as soy flour, tofu and soy sauce, have been consumed for more than 1000 years [23], while a new generation of soy food is prepared from soy isolates and concentrates. Functional and texturized soy protein concentrates with a protein content of 70% in dry matter are widely applied in processed meat products, owing to their functional properties, such as water binding, fat binding, texture and emulsification capability, providing improved economy with increased yields and a lower ingredient cost [24]. Thermal processing, high hydrostatic pressure treatments and fermentation can alter the structure of soy proteins and fragment DNA, depending on the conditions and duration of the processes. More details about the various steps of technological processing are summarized elsewhere [25,26]. Hence, a method for soybean as an allergen should allow for the detection of soy, most ideally independent from the various levels of processing, at a comparable sensitivity. Accordingly, eight soybean products processed to different degrees were investigated. All DNA and protein extracts were diluted 1:10^4^ prior to analysis to simulate low levels of soy (Table 2). The results obtained with the *ORF160b* LAMP-LFD were compared with those of the DNA-based *lectin* qPCR and two commercial protein-based LFDs (Table 2). As a control to avoid false-negative results in DNA-based analysis, the absence of amplification inhibitors and the integrity of the DNA were confirmed by a positive amplification of a conserved eukaryotic sequence on the *18S rRNA* gene in all samples.

Soybean DNA was detected in all investigated soybean products, when analyzed with the *ORF160b* LAMP-LFD and the *lectin* qPCR. However, in one TVP sample (P-2), not all replicates of qPCR or LAMP-LFD tested positive, which indicates a reduced sensitivity. Similarly, not all replicates tested positive in tofu using qPCR. The commercial protein-based LFD1 also detected soybean in all eight samples, which was consistent with the results of the DNA-based methods. By contrast, the commercial LFD2 tested negative in several samples, including TVP, soy flakes and tofu.

The results indicate that the DNA-based detection of soy products, using two different principles of specific DNA amplification (PCR and LAMP) is not critically impacted by soy processing under various conditions. By contrast, such processing conditions may lead to modifications of the protein structure, so that antigen-antibody binding can be negatively affected. As such, antibody-based detection of soy proteins might be negatively impacted by the loss of epitopes relevant for detection, as was assumed for LFD2.

### 3.3. Determination of Method Sensitvity in Soybean-Incurred Food Matrices

#### 3.3.1. Verification of Various Levels of Incurred Soybean

The sensitivity of methods was determined in model food matrices having incurred soybean at known levels. Prior to fortification with defined amounts of ground yellow soybean powder, the food matrices were first tested negative for soybean DNA using the *lectin* qPCR. Sausages were selected due to their high content of salt, fat and proteins, which can act as inhibitors of amplification reactions [27]. Chocolate as a matrix contains high amounts of fat, carbohydrates and phenolic compounds. Phenolic groups potentially denature proteins by binding to N-substituted amides or could oxidize to form a Quinone, which covalently binds to DNA, the polymerase and other proteins [27,28]. The soup was chosen as a food matrix having low pH, which could lead to denaturation of the double stranded DNA, the polymerase and other proteins.

**Figure 1:**
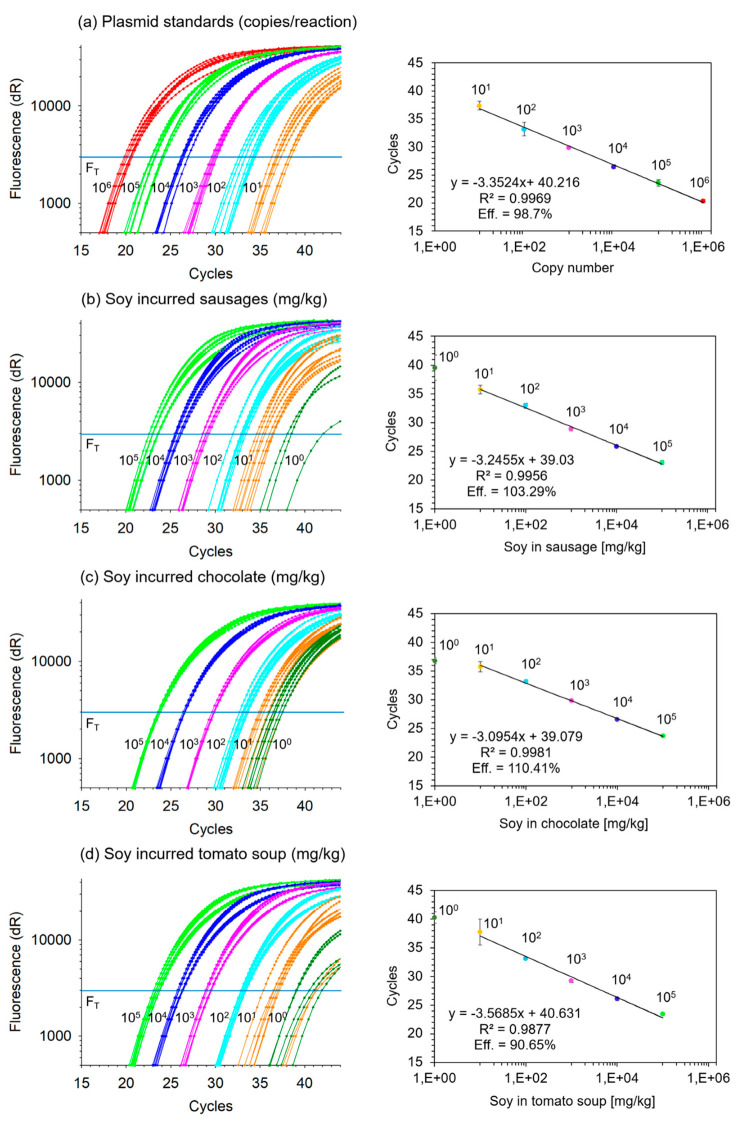
*Lectin* qPCR: Amplification curves (left panel) and linear regression (right panel) of fluorescence threshold (F_T_) cycles versus *lectin* copy numbers or mg/kg soy in matrix (**a**) plasmid DNA standards between 10^1^ and 10^6^ copies per reaction; (**b**) soybean incurred between 10^0^ and 10^5^ mg per kg sausage; (**c**) soybean incurred between 10^0^ and 10^5^ mg per kg chocolate; (**d**) soybean incurred between 10^0^ and 10^5^ mg per kg tomato soup. Each level of plasmid DNA standard or incurred soybean sample was analyzed in eight replicate reactions each (error bars indicate standard deviation of replicate means; R^2^: coefficient of determination; Eff: efficiency).

In order to verify the 10-fold serial grading levels between 10^0^ and 10^5^ mg/kg soybean in food matrix, each level was analyzed in eight replicates using the *lectin* qPCR (Table 3, Figure 1). The validity of each qPCR run was verified using 10-fold serial dilutions of standard plasmid DNA harboring the sequence of the 81 bp *lectin* amplicon. Figure 1a displays representative amplification curves and positions of threshold cycles of valid qPCR runs of plasmid DNA between 10^1^ and 10^6^ copies per reaction. Good linearity (R-squared, coefficient of determination) and PCR efficiency near 100% were obtained.

Figure 1b–d shows the PCR amplification curves of 10-fold-graded amounts of soybean in the matrices sausage, chocolate and instant tomato soup, respectively (Figure 1). Between 10^1^ and 10^5^ mg soybean per kg matrix, all replicates tested positive in qPCR. In addition, the level of 10^0^ mg soybean per kg matrix also tested positive; however, not in all replicates of the matrices sausage and tomato soup. By average, we obtained around 50% rate of positivity at the level of 10^0^ mg/kg soy in sausage or soup. This also corresponded well with approximately one copy of amplified DNA when comparing Figure 1a with Figure 1b–d. According to Poisson distribution, this rate of positivity may indicate slightly more than a single-copy detection. The lowest theoretical LOD with a 95% rate of positivity per PCR is three copies [29]. As a simplified approximation, this may indicate that the limit of detection of the *lectin* qPCR with 95% of positive samples is around the level of 3 mg/kg soybean in matrix. Accordingly, regression analysis was performed with data obtained from samples having between 10^1^ and 10^5^ mg soybean per kg matrix, without inclusion of data from the 10^0^ mg/kg level.

With a mean R-squared of 0.9938 ± 0.0054, the regression analysis of threshold cycle versus log of mg/kg soybean incurred in matrix achieved good linearity. Taking into account an average slope of −3.3031 ± 0.2418 (range from −3.0954 to −3.5685) in the regression equation, a 9.87 (2^3.3031^)-fold serial grading was obtained over the three matrices on average. Moreover, with a mean intercept of 39.59 ± 0.91 cycles over three matrices, corresponding to 10^0^ mg/kg soy in matrix, the grading levels were also very much comparable. In summary, a 10-fold serial grading of the matrices was confirmed for the levels ranging between 10^1^ and 10^5^ mg soybean per kg matrix. The lower level of 10^0^ mg/kg did not follow the linear regression in all matrices due to low numbers of lectin copies, but there was still indication of a likely correct level of incurred soybean. Hence, we concluded that the process of generating 10-fold serial gradings of soybean incurred into the food matrices sausage, chocolate and instant tomato soup worked successfully. The qualitative results are summarized in Table 3.

#### 3.3.2. Comparison of Sensitivity of ORF160b LAMP-LFD and Commercial Protein-Based LFDs

The matrices with verified levels between 10^0^ and 10^5^ mg incurred soybean per kg matrix were analyzed in each eight replicates using the *ORF160b* LAMP-LFD. Moreover, levels between 10^0^ and 10^3^ mg/kg were analyzed in each single replicate using both commercial protein-based LFDs. Blank matrices served as negative controls (Table 3). Down to the level of 10 mg/kg soybean in matrix (0.001%), all replicates of all three matrices tested positive using the DNA-based *ORF160b* LAMP-LFD assay. The level of 10^0^ mg/kg (0.0001%) soybean per matrix tested positive in all replicates of chocolate analysis, but not of sausage and tomato soup, respectively. Based on the analysis of three selected matrices, the sensitivities of *ORF160b* LAMP-LFD and qPCR appeared to be highly comparable, and superior to the protein-based LFDs. Using the protein-based LFD tests, all incurred matrices were tested positive down to a level of 10^2^ mg/kg (0.01%). Using LFD1, very faint test lines were observed at the 10 mg/kg level in sausage and the 10^0^ mg/kg level in the chocolate and tomato soup matrix, but also in the blank tomato soup matrix, which indicates a certain susceptibility to false positive signals in this matrix. Using LFD2, one of three matrices, namely chocolate tested negative at the level of 10 mg/kg (0.001%) (Table 3). McNemar’s Test, based on a 2 × 2 contingency table, was applied to test for statistical significance of differences between results of the detection methods, i.e., for LAMP versus (vs.) qPCR, LAMP vs. LFD1, LAMP vs. LFD2, and LFD1 vs. LFD2. Between LAMP and qPCR, no differences were observed that could be analyzed. Between LAMP and the LFDs, and between both LFDs, in all cases χ^2^ was below the critical value and *p* > 0.1, indicating no statistical significance of the observed differences, which also might be due to the limited number of analyzed samples (*n* = 15).

**Figure 2:**
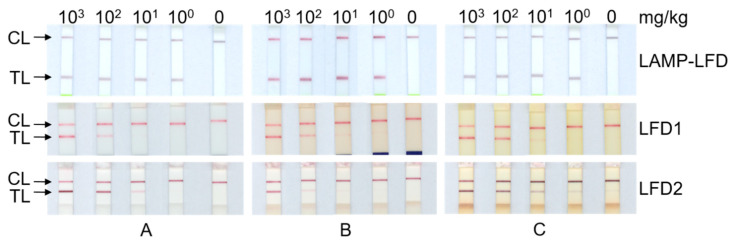
Comparison of test results of *ORF160b* LAMP-LFD, and both commercial protein-based LFD, of various levels of incurred soybean in matrix: (**A**) sausage; (**B**) chocolate; (**C**) tomato soup matrix. Digital recording of developed test strips resulted in final incubation times that exceeded the manufacturers’ recommendations, and partly led to unspecific detection of protein-based LFD. (CL: control line; TL: test line).

In addition, Figure 2 displays the results of *ORF160b* LAMP-LFD, and both protein-based LFDs after extended final incubation for the purpose of digital documentation (Figure 2).

The manual of LFD2 indicates that the test may be prone to the development of false positive results when extending the 5-min incubation period. Despite the exact incubation for 5 min and immediate removal of test stripes from the incubation mixture, test lines of incurred tomato soup at the 10^0^ mg/kg level and of the blank matrix developed slight positive signals (Figure 2C), while formerly being negative at the visual inspection immediately after the 5 min incubation time (Table 3). In contrast to results of the protein-based LFDs, where only faint test lines were visible at low soybean levels, the color intensity of the test line of the DNA-based LAMP-LFD assay was independent of the initial level of soybean in the matrices tested (Figure 2). In addition, the results of the DNA-based LAMP-LFD were stable and did not change even after extended storage for several days. Hence, the *ORF160b* LAMP-LFD allows an unambiguous assessment of test results in comparison to dose- and time-dependent signals in antibody-based LFDs at low levels of detected soybean.

### 3.4. Analyses of Retail Food Samples

Twelve commercial retail foods were selected with regard to a broad spectrum of matrices rich in carbohydrates, proteins, fats and other components known to potentially inhibit DNA amplification reactions (Table 4). We selected three products without soybean, according to the list of ingredients or PAL statements, two products with only PAL statements and seven products containing soybean, according to the ingredient list. The results obtained with the *ORF160b* LAMP-LFD were compared with those of the *lectin* qPCR and the two investigated commercial antibody-based LFD tests. In general, the results obtained with both DNA-based and protein-based methods were in good agreement, apart from some foods in which soybean was interpreted to be present around the LOD of the methods (Table 4). The detailed results are discussed as follows.

Using the *ORF160b* LAMP-LFD, all replicates tested positive in six (R-1, R-3-5, R-7-8) of the seven samples that were declared to contain soybean. Using the qPCR and commercial LFD1, six of the seven samples were also, at least partly, tested soybean positive, while, using the commercial LFD2, five of the seven samples tested positive. In contrast to the reproducible positive results obtained using LAMP-LFD, the milk chocolate with puffed rice (R-7), containing soy lecithin as emulsifier, tested negative using commercial LFD2 and only tested weakly positive using the commercial LFD1. Using qPCR, in average 2.6 ± 1.3 copies of genomic soybean DNA were quantified in some of the replicates, which is around the LOD of the method. Soy lecithin is used as stabilizer and emulsifier in a wide range of foods. Soy lecithin is mostly obtained by hexane extraction during the manufacturing of soy oil. Lecithins are complex mixtures, which also contain residual proteins in variable amounts depending on the manufacturing process. Proteins present in lecithin may trigger allergic reactions in sensitive individuals [30].

**Figure 3:**
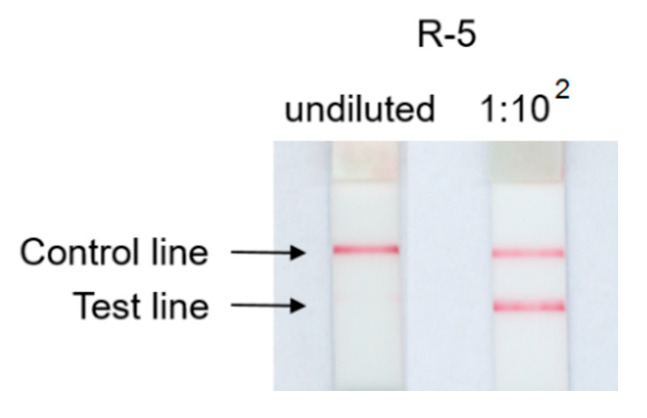
Hook effect observed in LFD1: Retail food R-5 tested negative from an undiluted sample extract, but positive after 1:10^2^ extract dilution.

The meat free, lactose free sausage-type bar for barbecue (R-5), containing 12% soy protein, initially tested negative in the commercial LFD1. Further dilution of the protein extract at 1:10^2^ resulted in a positive test line (Figure 3). This so-called hook effect occurs when both the detection antibodies and the capture antibodies are saturated with analyte and the antibody-analyte-antibody sandwich formation is impeded [31]. Thus, LFDs may be susceptible to false negative results when a large amount of the target analyte, i.e., allergenic soybean in this study, is present. Such a hook effect was not observed using the *ORF160b* LAMP-LFD that presented reproducible and clearly visible positive test and control lines (data not shown).

The Asia style rice crackers (R-2), declared to contain soy sauce, were tested negative for soybean in both DNA-based amplification methods and protein-based LFDs. One explanation might be DNA fragmentation and protein hydrolyzation caused by extensive processing as is done to manufacture soy sauce.

Of the two samples, having PAL of soybean, the hazelnut cookies (R-9), tested negative using both commercial protein-based LFDs, while the DNA-based methods tested positive for soybean in some of the analyzed replicates. Moreover, in qPCR, an average of 0.7 ± 0.3 copies was determined in the positive replicates, which is below the LOD of the method. Despite of soybean PAL in the oat porridge sample (R-10), neither DNA-based amplification methods nor protein-based LFDs tested positive for soybean. Against a missing soybean declaration in the ingredient list or using PAL, *ORF160b* LAMP-LFD and qPCR, tested positive in all replicates of the sample ‘cereal bar with chocolate’ (R-6). In this food, the commercial protein-based LFD1 showed a weakly positive test line, while LFD2 tested negative. Similarly, the cereal-potato-snack with Western style flavor (R-12), which did not have soybean declared as ingredient or according to PAL, the DNA-based LAMP-LFD tested negative, while the qPCR tested positive in some replicates, of which an average of 1.1 ± 1.6 copies per replicate were determined as being below the LOD. Similarly, the protein-based LFD1 showed a faint positive test line, while the commercial LFD2 tested negative. Compliant to the negative declaration, the roasted and salted cashews (R-11) tested negative for soybean using both the DNA-based amplification methods and the protein-based LFDs. The DNA-based methods showed partly positive amplification signals for the hazelnut cookies (R-9), whereas both commercial LFDs tested negative.

Table 4 shows that the numbers of positive and negative results from the analysis of these samples correlate closely between the DNA-based amplification methods, *lectin* qPCR and the *ORF160b* LAMP-LFD assay, and both commercial protein-based LFDs. In accordance with the results shown for the matrices with incurred soybean (Table 3), the results from retail samples (Table 4) once more indicate that in some cases the DNA-based methods appear to be more sensitive than the protein-based LFDs. McNemar’s Test was applied to test for statistical significance of these differences, i.e., for LAMP vs. qPCR, LAMP vs. LFD1, LAMP vs. LFD2, and LFD1 vs. LFD2. In all cases, χ^2^ was below the critical value and *p* > 0.1, indicating no statistical significance of the observed differences, which also might be due to the limited number of analyzed samples (*n* = 12).

Taken together, these results suggest that the *ORF160b* LAMP-LFD is comparably sensitive but more rapid as the *lectin* qPCR. Moreover, as the results of *ORF160b* LAMP-LFD are in good agreement with the protein-based LFDs, the isothermal LAMP-LFD assay is considered an alternative qualitative method to protein-based LFD.

## 4. Conclusions

Considering the published reference dose of 0.5 mg soybean protein [7], obtained from clinical studies, as the safe level for the great majority of soybean allergic subjects not to experience an allergic reaction, a detection method would need to verify soybean at such low level. To reach this amount of soybean reference protein, the soybean protein concentration in the food matrix and the ingested portion size need to be considered. Taking 100 g of food as an example for a medium sized portion, the sensitivity of a soybean detection method would require verification of 5 mg soybean protein or 12.5 mg soy per kg food, on the basis of 40% soybean protein as an average [32]. Previously, we described the development of primers for the detection of soy using loop-mediated isothermal amplification (LAMP), combined with a lateral-flow dipstick (LFD) detection of amplicons of *ORF160b* mitochondrial soybean DNA. The primers proved highly specific for soybean and allowed for the detection of a range of soybean cultivars [18]. Here, we investigated the sensitivity of this *ORF160b* LAMP-LFD to detect soybean in a range of food matrices and in comparison, the state-of-the-art qPCR and commercial protein-based LFD.

With a sensitivity at or below 10 mg soybean per kg food matrix, the DNA-based *ORF160b* LAMP-LFD proved sensitive enough, while the sensitivity of the investigated commercial protein/antibody-based LFDs ranged approximately between 10 and 10^2^ mg/kg, depending on the matrix and test used. Between four and eight detection replicates were analyzed using the DNA-based methods, qPCR and LAMP, to identify the level of reproducible detection. At a level of 10 mg/kg soybean in various food matrices, reproducible detectability was found. Hence, for routine use and screening purposes, the number of replicates may be reduced. Overall, the *ORF160b* LAMP-LFD proved a sensitive, specific and low-tech DNA-based method for soybean detection, which is as an alternative to state-of-the-art qPCR. With less than one hour of testing, the LAMP-LFD showed further potential as a rapid DNA-based detection tool for allergens and, as an alternative method for rapid protein-based LFD. Prior to using LAMP-LFD as a full alternative to rapid antibody-based methods, the extraction procedure to obtain amplifiable DNA would require additional attention, especially regarding ease and speed. At this stage, the *ORF160b* LAMP-LFD represents a rapid and simple screening tool based on extracted DNA prior to potential quantification of positives with qPCR techniques. Once further optimized for speedy DNA extraction and amplification, the presented LAMP-LFD can potentially replace antibody-based rapid methods.

Advantages of this highly sensitive, soy specific DNA-based LAMP-LFD over commercial antibody-based LFD for soy detection are the clearly visible and stable test and control lines and no susceptibility to hook effects that we observed in antibody-based LFD. Moreover, the complete chemistry of LAMP-LFD is defined, disclosed and open access, which might present mandatory criteria for governmental food control laboratories. No allergen-specific antibodies are required, which eliminates the necessity of repetitive animal immunization experiments to regain polyclonal antibodies, once they become scarce.

## Figures and Tables

**Table 1:** Number of replicate analysis according to type of investigation and method used.

Investigation	*ORF160b* LAMP-LFD	*Lectin* qPCR	Commercial LFD ^1^
Specificity	Allgöwer et al. 2020 [18]	n.d. ^2^	1 rxn ^3^, 1:10 dilution (1 extract × 1 rxn)
Processed soy products	4 rxns, 1:10^4^ dilution (2 extracts × 2 rxns)	4 rxns, 1:10^4^ dilution (2 extracts × 2 rxns)	1 rxn, 1:10^4^ dilution (1 extract × 1 rxn)
Sensitivity in incurred food matrices	8 rxns, undiluted (2 extracts × 4 rxns)	8 rxns, undiluted (2 extracts × 4 rxns)	1 rxn, undiluted (1 extract × 1 rxn)
Retail foods	4 rxns, undiluted (2 extracts × 2 rxns)	4 rxns, undiluted (2 extracts × 2 rxns)	1 rxn, undiluted (1 extract × 1 rxn)

^1^ No. of replicates (reactions, rxns), according to the manufacturer’s instructions; ^2^ n.d.: not determined; ^3^ rxn(s): reaction(s).

**Table 2:** Detection of processed soy products analyzed at 1:10^4^ dilution using loop-mediated isothermal amplification (LAMP)-lateral flow dipstick (LFD), quantitative real-time PCR (qPCR) and two commercial LFD tests. (+: all replicates positive; −: all replicates negative; (+): LAMP/qPCR: not all replicates positive or negative).

Sample No.	Soy Product	Protein Content (%)	*ORF160b* LAMP-LFD	*lectin* qPCR	LFD1	LFD2
P-1	TVP ^1^	56.7	+	+	+	−
P-2	TVP ^1^	67.4	(+)	(+)	+	−
P-3	soy flakes	~50 ^2^	+	+	+	−
P-4	soy flakes	~50 ^2^	+	+	+	+
P-5	soy protein concentrate	71.8	+	+	+	+
P-6	defatted soy flour	~50 ^2^	+	+	+	+
P-7	soy semolina	~50 ^2^	+	+	+	+
P-8	tofu	13.5	+	(+)	+	−
18	Yellow soybeans Schoenenberger^®^ Hensel^®^	38	+	+	+	+

^1^ TVP: texturized vegetable protein; ^2^ actual protein content unknown, protein content assumed based on literature (CODEX STAN 175, 1989); samples P-1 to P-7 from ifp, P-8 from Life Food GmbH.

**Table 3:** Qualitative results from the analysis of soybean incurred in food matrix using qPCR, LAMP-LFD, and two commercial LFD tests, strictly according to the manufacturers’ instructions. (+: all replicates positive; −: all replicates negative; (+): not all replicates of LAMP/qPCR tested positive, or faint test line in commercial LFD).

Method	Food Matrix	Level of Incurred Soybean per Matrix (mg/kg)						
		10^5^	10^4^	10^3^	10^2^	10^1^	10^0^	0 ^1^
*lectin* qPCR	sausage	+	+	+	+	+	(+)	−
	chocolate	+	+	+	+	+	+	−
	tomato soup	+	+	+	+	+	(+)	−
*ORF160b* LAMP-LFD	sausage	+	+	+	+	+	(+)	−
	chocolate	+	+	+	+	+	+	−
	tomato soup	+	+	+	+	+	(+)	−
LFD1	sausage	n.d. ^2^	n.d.	+	+	(+)	−	−
	chocolate	n.d.	n.d.	+	+	+	(+)	−
	tomato soup	n.d.	n.d.	+	+	+	(+)	(+)
LFD2	sausage	n.d.	n.d.	+	+	+	−	−
	chocolate	n.d.	n.d.	+	+	−	−	−
	tomato soup	n.d.	n.d.	+	+	+	−	−

^1^ 0: blank matrix; ^2^ n.d.: not determined.

**Table 4:** Analysis of retail foods (R) for soybean using *ORF160b* LAMP-LFD and two commercially available protein-based LFDs. The *lectin* qPCR served as a comparative DNA-based detection method (+: all replicates tested positive; −: all replicates tested negative; (+): not all replicates of LAMP/qPCR tested positive, or faint test line in commercial LFD).

Sample No.	Product Description	Soy: Ingredient Labeling (IL)/Precautionary Allergen Labeling (PAL)	Interpretation of IL/PAL	*Lectin* qPCR	*ORF160b* LAMP-LFD	LFD1	LFD2
R-1	fine biscuit assortment	IL: soy flour, emulsifier: soy lecithin/PAL: none	contains soy	+	+	+	+
R-2	Asia style rice crackers	IL: soy sauce (soybeans, …)/PAL: contains soy	contains soy	−	−	−	−
R-3	sponge cake vanilla and raspberry taste with cocoa compound coating	IL: soybean, soy flour, emulsifier: soy lecithin/PAL: contains soy	contains soy	+	+	+	+
R-4	vegetable balls	IL: 10% plant protein (soy protein, wheat protein), hydrolyzed soy protein/PAL: none	contains soy	+	+	+	+
R-5	meat free, lactose free sausage-type bar for barbecue	IL: 12% soy protein/PAL: none	contains soy	+	+	± ^1^	+
R-6	cereal bar with chocolate	IL: none/PAL: none	no indication of soy presence	+	+	(+)	−
R-7	milk chocolate with puffed rice	IL: emulsifier: soy lecithin/PAL: none	contains soy	(+)	+	(+)	−
R-8	shortbread with milk chocolate and chopped almonds	IL: soy flour, emulsifier: soy lecithin/PAL: none	contains soy	+	+	+	+
R-9	hazelnut cookies	IL: none/PAL: produced in a facility, where soy is also processed	may contain soy	(+)	(+)	−	−
R-10	oat porridge	IL: none/PAL: may contain traces of soy	may contain soy	−	−	−	−
R-11	cashews roasted and salted	IL: none/PAL: none	no indication of soy presence	−	−	−	−
R-12	Cereal-potato-snack Western style flavor	IL: none/PAL: none	no indication of soy presence	(+)	−	(+)	−

^1^ ±: negative test line in undiluted sample, but positive test line in 1:10^2^ diluted sample.

## Supplementary Materials

The following are available online at https://www.mdpi.com/2304-8158/9/12/1741/s1, Table S1: Specificity testing of two commercial protein-based LFD tests compared to *ORF160b* LAMP-LFD. Table S2: Primers for *ORF160b* LAMP, *lectin* qPCR and eukaryotic *18S rRNA* qPCR used in this study.
