# Supplementary material for: LAMP-LFD Based on Isothermal Amplification of Multicopy Gene ORF160b: Applicability for Highly Sensitive Low-Tech Screening of Allergenic Soybean (Glycine max) in Food
Source: Foods. 2020 Nov 26;9(12):1741. doi: 10.3390/foods9121741 (PMC7760099; doi:10.3390/foods9121741)
Supplement: Supplementary file 1 [file foods-09-01741-s001.pdf]

foods

Supplementary material

LAMP-LFD Based on Isothermal Amplification of Multicopy Gene *ORF160b*: Applicability for Highly Sensitive Low-tech Screening of Allergenic Soybean (*Glycine max*) in Food

Stefanie M. Allgöwer, Chris A. Hartmann, Clarissa Lipinski, Vera Mahler, Stefanie Randow, Elke Völker, and Thomas Holzhauser *

Division of Allergology, Paul-Ehrlich-Institut (PEI), D-63225 Langen, Germany;
stefanie.allgöwer@arcor.de (S.M.A.); chris.a.hartmann@gmx.de (C.A.H.); dr.clarissa.lipinski@gmail.com (C.L.); vera.mahler@pei.de (V.M.); stefanie.randow@pei.de (S.R.); elke.völker@pei.de (E.V.);
* Correspondence: thomas.holzhauser@pei.de; Tel.: +49-6103-77-5304 (T.H.)

Table S1. Specificity testing of two commercial protein-based LFD tests compared to *ORF160b* LAMP-LFD. Yellow soybean served as positive control (+: positive test line; -: negative test line).

Sample No.	Common Name	Species Name	Family	*ORF160b* LAMP-LFD [3]	LFD1	LFD2
1	peanut [1,2]	*Arachis hypogaea*	*Fabaceae*	-	-	-
2	sesame seed [1]	*Sesamum indicum*	*Pedaliaceae*	-	-	-
3	cow's milk [1]	*Bos taurus*	*Bovidae*	-	-	-
4	oat [1]	*Avena sativa*	*Poaceae*	-	-	-
5	barley [1]	*Hordeum vulgare*	*Poaceae*	-	-	-
6	rye [1]	*Secale cereale*	*Poaceae*	-	-	-
7	wheat [1]	*Triticum sp.*	*Poaceae*	-	-	-
8	brown lentil [2]	*Lens culinaris*	*Fabaceae*	-	-	-
9	pea [2]	*Pisum sativum*	*Fabaceae*	-	-	-
10	white mustard seed [1]	*Sinapis alba*	*Brassicaceae*	-	-	-
11	blue lupine [1,2]	*Lupinus angustifolius*	*Fabaceae*	-	-	-
12	yellow lupine [1,2]	*Lupinus luteus*	*Fabaceae*	-	-	-
13	white lupine [1,2]	*Lupinus albus*	*Fabaceae*	-	-	-
14	hazelnut [1]	*Corylus avellana*	*Betulaceae*	-	-	-
15	spelt [1]	*Triticum aestivum* subsp. *spelta*	*Poaceae*	-	-	-
16	squid [1]	*Loligo formosana*	*Loliginidae*	-	-	-
17	celeriac [1]	*Apium graveolens* var. *rapaceum*	*Apiaceae*	-	-	-
18	soybean [1,2]	*Glycine max*	*Fabaceae*	+	+	+

[1] require mandatory labeling as ingredient according to EU Directive 1169/2011; [2] legume (*Fabaceae*); [3] previously published [18].

Table S2. Primers for *ORF160b* LAMP, *lectin* qPCR and eukaryotic *18S rRNA* qPCR used in this study.

Target Gene	primer name	sequence (5'→3')
ORF160b [1]	F3 ORF160b	CCGAGTCTGCTGCCGTAT
	B3 ORF160b	ATGAGATTGAGTTCCACGCA
	FIP ORF160b	GGGGTCAGTATTACGCCTCTGACAAAGAAAGAGAGTGACGATG
	biotin-FIP ORF160b	biotin-GGGGTCAGTATTACGCCTCTGACAAAGAAAGAGAGTGACGATG
	BIP ORF160b	TCTGATAGATAGTGGCAAACATTAGTTGCTGCTATTCCATCTATTCAT
	LoopF ORF160b	TTCTGATTCCGCTCATTGG
	FITC-LoopF ORF160b	FITC-TTCTGATTCCGCTCATTGG
	LoopB ORF160b	CAAGATATAGAAGACTATTAGCCCG
Lectin [2]	Lectin-F	TCCACCCCCATCCACATTT
	Lectin-R	GGCATAGAAGGTGAAGTTGAAGGA
	Lectin-probe	ROX-AACCGGTAGCGTTGCCAGCTTCG-BBQ-650
18S rRNA [3]	TR03 (forward)	TCTGCCCTATCAACTTTCGATGGTA
	TR04 (reverse)	AATTTGCGCGCCTGCTGCCTTCCTT

[1] [18]; [2] [20]; [3] [21].

References

[18] Allgöwer, S.M.; Hartmann, C.A.; Holzhauser, T. Sensitive detection of allergenic soybean (Glycine max) using multicopy gene loop-mediated isothermal amplification combined with lateral flow dipstick (LAMP-LFD). *Foods* **2020**, *9(4):423*, 2-19.

[20] BVL Official collection of Test methods. Detection and quantification of mustard (Sinapis alba) and soybean (Glycine max) in boiled sausages with real-time PCR. *German Food and Feed Law-Food Analysis* **2012**, *Article 64, L 08.00-59*, 1-15.

[21] Allmann, M.; Candrian, U.; Höfelein, C.; Lüthy, J. Polymerase chain reaction (PCR): a possible alternative to immunochemical methods assuring safety and quality of food, Detection of wheat contamination in non-wheat food products, *Z Lebensm Unters Forsch* **1993**, *196(3)*, 248-251.

© 2020 by the authors. Submitted for possible open access publication under the terms and conditions of the Creative Commons Attribution (CC BY) license (http://creativecommons.org/licenses/by/4.0/).
